# Enhanced nattokinase production by *Bacillus subtilis* from glycerol and okara: optimization of culture medium via response surface methodology

**DOI:** 10.3389/fmicb.2025.1577292

**Published:** 2025-05-14

**Authors:** Yaling Duan, WanTing Du, Yang Xu, Gege Guo, Zhaoxing Liu, Ning Hao

**Affiliations:** State Key Laboratory of Materials-Oriented Chemical Engineering, College of Biotechnology and Pharmaceutical Engineering, Jiangsu National Synergetic Innovation Center for Advanced Materials (SICAM), Nanjing Tech University, Nanjing, China

**Keywords:** nattokinase, glycerol, okara, *Bacillus subtilis*, fermentation

## Abstract

**Introduction:**

Nattokinase is an alkaline serine protease with potent thrombolytic activity. Due to its safety profile, low cost, and ease of oral administration, it has gained significant attention for therapeutic applications. To meet the demand for cost-effective production of nattokinase via fermentation, this study utilized renewable biomass resources—glycerol and okara—as raw materials for nattokinase production.

**Methods:**

A metabolic pathway for efficient glycerol utilization in Bacillus subtilis 13,932 was engineered using genetic modifications. The co-expression of glpF and glpK significantly enhanced the strain’s ability to metabolize glycerol. Building upon this, fermentation conditions using glycerol as the carbon source and okara as the nitrogen source were optimized. A Box-Behnken Design was employed to determine the optimal medium composition. Additionally, fermentation parameters were further optimized through a single-factor experiment.

**Results:**

The optimal medium composition was determined to be: glycerol 23 g/L, okara 96 g/L, MgSO_4_⋅7H_2_O 0.8 g/L, and CaCl_2_ 0.7 g/L, yielding a nattokinase activity of 8709.53 ± 103.45 IU/mL. Further optimization of fermentation parameters resulted in the highest nattokinase activity of 10576.28 ± 91.78 IU/mL under the following conditions: 37 °C, 200 rpm shaking speed, 7% inoculum, and an initial pH of 7.5.

**Discussion:**

The genetic modifications enabling efficient glycerol metabolism in Bacillus subtilis 13,932, along with the optimized fermentation strategy using renewable resources, significantly improved nattokinase production. The high enzymatic activity achieved demonstrates the potential of this approach for cost-effective and sustainable nattokinase production.

## 1 Introduction

Microbial fermentation of renewable biomass feedstocks for the production of enzymes and various chemicals offers a promising strategy for maximizing the value of these materials (Khunnonkwao et al., 2024). Among these feedstocks, glycerol stands out as a significant by-product of bioethanol and biodiesel production, with 1 to 1.4 tons of crude glycerol generated for every 10 tons of biodiesel produced (Elsayed et al., 2024; Zhang et al., 2016). As the biofuel industry continues to grow, the output of crude glycerol has been steadily increasing, with projections suggesting it could reach 7.66 million tons by 2022, while prices remain below $100 per ton (Zhao et al., 2023). Given that crude glycerol has a value comparable to lignocellulosic hydrolysates used in fuel ethanol production, converting glycerol into high-value products via biotransformation could substantially improve the economic sustainability of the biodiesel industry (Kumar et al., 2019).

Glycerol, as a renewable biomass resource, not only holds promise as an alternative carbon source for biofuel production (Murarka et al., 2008), but also serves as a valuable feedstock for the production of high-value products such as nattokinase. Nattokinase, a fibrinolytic protease with potent thrombolytic activity, has gained attention due to its medical applications, particularly for cardiovascular diseases (Cai et al., 2017; Wu et al., 2019). Given the rising demand for affordable and safe nattokinase, enhancing its production efficiency through microbial fermentation using low-cost substrates like glycerol becomes increasingly important. Several studies have explored the use of low-cost substrates for nattokinase fermentation (Li et al., 2022; Sahoo et al., 2020; Wang et al., 2009), with *B. subtilis natto* as a popular host. Researchers have successfully employed substrates such as soybean milk, tofu processing wastewater, and hemp seed meal to achieve high enzymatic activity (Li et al., 2021; Nie et al., 2017; Zhang et al., 2023). Moreover, renewable biomass materials, including glycerol, cheese whey wastewater, and okara, have shown significant potential in nattokinase fermentation. Previous studies have primarily relied on traditional carbon sources (e.g., glucose) and nitrogen sources (e.g., soybean peptone) for nattokinase production. However, these raw materials are not only costly but also lack sustainability. Although some studies have explored the potential of low-cost substrates such as glycerol, significant research gaps remain in optimizing glycerol utilization through metabolic engineering to enhance nattokinase yield.

To address these limitations, this study fills the existing research gaps through the following innovative approaches: First, glycerol, a byproduct of biodiesel production, and okara, a waste product from tofu processing, were employed as carbon and nitrogen sources, respectively, significantly reducing production costs and improving resource sustainability. Second, the overexpression of key glycerol metabolic genes (*glpF* and *glpK*) through metabolic engineering significantly enhanced glycerol utilization efficiency, thereby increasing nattokinase yield. Finally, systematic optimization of fermentation conditions was achieved using response surface methodology and single-factor experiments, further improving nattokinase activity. These innovations not only effectively address the high costs associated with traditional production methods but also provide a new technical pathway for efficient enzyme production using renewable resources.

## 2 Materials and methods

### 2.1 Materials and reagents

Fibrinogen (70 mg/bottle), thrombin (1,180 Units/bottle), and urokinase (1,560 IU/bottle) were purchased from the China Institute for Food and Drug Control (Beijing, China). Agarose was purchased from Sigma-Aldrich (St. Louis, Missouri, United States), and other analytical reagents were purchased from Nanjing Wanqing Chemical Instrument Co., Ltd. (Nanjing, China). The bean dregs were obtained from Nanjing Fruit Food Co., Ltd. After drying at 60°C, the bean dregs were crushed and sieved using a 40-mesh sieve.

### 2.2 Medium and culture conditions

Seed Liquid Medium: 10 g/L tryptone, 5 g/L yeast extract, and 10 g/L sodium chloride. Basic Medium: 20 g/L glucose, 20 g/L soy peptone, 1 g/L Na_2_HPO_4_, 1 g/L NaH_2_PO_4_, 0.2 g/L CaCl_2_, 0.6 g/L MgSO_4_⋅7H_2_O, 0.5 g/L ZnSO_4_. Glycerol Fermentation Medium: The carbon source in the basic medium was replaced with glycerol, while the remaining components remained unchanged. A ring of bacterial inoculum was introduced into 100/500 mL seed liquid medium, cultured overnight at 37°C and 200 rpm for 14–16 h, and then inoculated into glycerol fermentation medium at a 5% (v/v) inoculation rate until the end of fermentation.

### 2.3 Strains, plasmids and gene manipulation

*B. subtilis* 13,932 was preserved in the General Microbiology Center of the China Microbiological Culture Collection and Management Committee, with preservation number CGMCC No. 13932. The shuttle plasmid pMA5 was purchased from Wuhan Miaoling Biotechnology Co., Ltd. The strain, plasmid and primers used in this study is shown in Tables 1, 2.

**TABLE 1:** Strains and plasmids.

Strains and plasmids	Description	Source
**Strains**		
*E. Coli* DH5α	deoR endA1 gyrA96 hsdR17 (rk-mk +) recA1 relA1 supE44 thi-1 Δ(lacZYA-argF) U169 Φ80lacZ ΔM15F - λ -	Vazyme Biotech Co.,Ltd
*B. subtilis* 13932	Expression host (isolated from Chinese tempeh and subsequently preserved in the General Microbiology Center of the Chinese Microbial Culture Collection and Management Committee, accession number CGMCC No. 13932)	Laboratory storage
BS01	*B. subtilis* 13932 contains plasmid pMA5-*glpF*	This study
BS02	*B. subtilis* 13932 contains plasmid pMA5-*glpK*	This study
BS03	*B. subtilis* 13932 contains plasmid pMA5-*glpP*	This study
BS04	*B. subtilis* 13932 contains plasmid pMA5-*glpD*	This study
BS05	*B. subtilis* 13932 contains plasmid pMA5-*tipA*	This study
BS06	*B. subtilis* 13932 contains plasmid pMA5-*glpF*-*glpK*	This study
**Plasmids**		
pMA5	*Bacillus subtilis*-*E. coli* shuttle plasmid containing the *P_*HpaII*_* promoter	Purchased from Wuhan Miaoling Biotechnology Co., Ltd
pMA5-*glpF*	The *glpF* gene was constructed into the MCS region of the pMA5 plasmid	This study
pMA5-*glpK*	The *glpK* gene was constructed into the MCS region of the pMA5 plasmid	This study
pMA5-*glpD*	The *glpD* gene was constructed into the MCS region of the pMA5 plasmid	This study
pMA5-*glpP*	The *glpP* gene was constructed into the MCS region of the pMA5 plasmid	This study
pMA5-*tipA*	The *tipA* gene was constructed into the MCS region of the pMA5 plasmid	This study
pMA5-*glpF*-*glpK*	The *glpF*, *glpK* gene was constructed into the MCS region of the pMA5 plasmid	This study

**TABLE 2:** Primers.

Primer	Sequence (5′–3′)
*glpF*-F	aaagtgaaatcagggggatccatgacagcattttggggagaag
*glpF*-R	atttcgacctctagaacgcgtttaaatatatttagaatttgataatgttttagca
*glpK*-F	aaagtgaaatcagggggatccatggaaacgtacattttatccttagatc
*glpK*-R	atttcgacctctagaacgcgtttatttaaaagccatagctgctttca
*glpD*-F	aaagtgaaatcagggggatccatgatgaatcatcaattttcaagtctt
*glpD*-R	atttcgacctctagaacgcgtttattgctcaagcggtacgacc
*glpP*-F	aaagtgaaatcagggggatccatgatgagttttcacaaccagcc
*glpP*-R	atttcgacctctagaacgcgttcaatcactttccgtcaaaaagtt
*tpiA*-F	aaagtgaaatcagggggatccatgagaaaaccaattatcgccg
*tpiA*-R	atttcgacctctagaacgcgtttactcatattgaccttcctccaataa
*glpF-glpK*-F1	aaagtgaaatcagggggatccatgacagcattttggggagaag
*glpF-glpK*-R1	gataaaatgtacgtttccatttaaatatatttagaatttgataatgttttagca
*glpF*-*glpK*-F2	atggaaacgtacattttatccttagatc
*glpF*-*glpK-*R2	atttcgacctctagaacgcgtttatttaaaagccatagctgctttca

The genome of *B. subtilis* 168 was extracted using a bacterial genome extraction kit (Tiangen, China), and the target gene was amplified with the high-fidelity PCR enzyme 2 × Phanta Max Master Mix (Novozymes, China). The vector pMA5 was linearized using the restriction endonucleases *Bam*HI and *Mlu*I, and the vector and gene fragment were ligated using a one-step cloning kit (Novozymes, China) to construct a recombinant plasmid. The transformation method for *B. subtilis* was based on the *Spizizen* method (Anagnostopoulos and Spizizen, 1961; Wilson and Bott, 1968).

### 2.4 BBD optimization experiment

The effects of glycerol, okara, MgSO_4_⋅7H_2_O, and CaCl_2_ on the formation of nattokinase were optimized using a four-factor, three-level Box-Behnken design (BBD). The experimental design is shown in Table 3.

**TABLE 3:** Experimental design and results of Box-Behnken’s Design.

Run	A: glycerol (g/L)	B: okara (g/L)	C: MgSO_4_⋅7H_2_O (g/L)	D: CaCl_2_ (g/L)	Nattokinase activity (IU/mL)
1	10	60	0.75	0.55	5338.26 ± 104.32
2	35	60	0.75	0.55	6649.57 ± 93.46
3	10	100	0.75	0.55	6394.32 ± 84.47
4	35	100	0.75	0.55	7364.27 ± 123.75
5	22.5	80	0.5	0.1	7638.78 ± 102.54
6	22.5	80	1	0.1	8034.61 ± 116.32
7	22.5	80	0.5	1	7949.83 ± 103.68
8	22.5	80	1	1	8172.57 ± 76.49
9	10	80	0.75	0.1	5955.28 ± 107.26
10	35	80	0.75	0.1	7263.87 ± 84.59
11	10	80	0.75	1	6426.93 ± 93.46
12	35	80	0.75	1	6898.64 ± 106.92
13	22.5	60	0.5	0.55	7565.19 ± 102.63
14	22.5	100	0.5	0.55	8384.39 ± 122.75
15	22.5	60	1	0.55	7796.78 ± 107.33
16	22.5	100	1	0.55	8593.29 ± 98.48
17	10	80	0.5	0.55	5855.63 ± 105.95
18	35	80	0.5	0.55	7026.37 ± 104.74
19	10	80	1	0.55	6163.85 ± 89.83
20	35	80	1	0.55	7369.47 ± 103.57
21	22.5	60	0.75	0.1	7856.93 ± 103.15
22	22.5	100	0.75	0.1	8420.14 ± 117.87
23	22.5	60	0.75	1	8067.36 ± 108.52
24	22.5	100	0.75	1	8541.52 ± 96.54
25	22.5	80	0.75	0.55	8369.56 ± 112.38
26	22.5	80	0.75	0.55	8673.49 ± 131.58
27	22.5	80	0.75	0.55	8508.26 ± 86.26
28	22.5	80	0.75	0.55	8384.28 ± 114.95
29	22.5	80	0.75	0.55	8610.57 ± 105.09

### 2.5 Optimization of culture conditions

A single-factor experiment was conducted to further optimize the culture conditions based on the optimized medium. The recombinant strain BS06 was fermented in 100/500 mL glycerol fermentation medium for 72 h. The effects of shaking speed (120, 140, 160, 180, 200, and 220 rpm), inoculum size (v/v) (1%, 3%, 5%, 7%, 9%, and 11%), initial pH (5.5, 6.0, 6.5, 7.0, 7.5, and 8.0), and temperature (22, 27, 32, 37, 42, and 47°C) on nattokinase production by the recombinant strain BS06 were evaluated.

### 2.6 Analysis method

The UV spectrophotometer was used to determine the maximum absorbance at OD_600_, with stem cell weight calculated as 0.297 × OD_600_. Glycerol content was determined using an Agilent 1,260 high-performance liquid chromatograph. The chromatographic column used was Aminex HPX-87H, with an injection volume of 20 μL. The mobile phase consisted of 5 mM H2SO4, the flow rate was 0.6 mL/min, the column temperature was set at 65°C, and the temperature of the differential detector was 45°C (Rathnasingh et al., 2016). The activity of nattokinase was determined using the fibrin plate method, following the research of Li et al. (2020) 2021.

### 2.7 Statistical analysis

The data in this study are expressed as the mean ± standard deviation of three replicates from independent experiments. A *P*-value of < 0.05 indicates a significant difference, while a *P*-value of < 0.0001 indicates an extremely significant difference.

## 3 Results

### 3.1 Fermentation of nattokinase activity by different substrates

**FIGURE 1:**
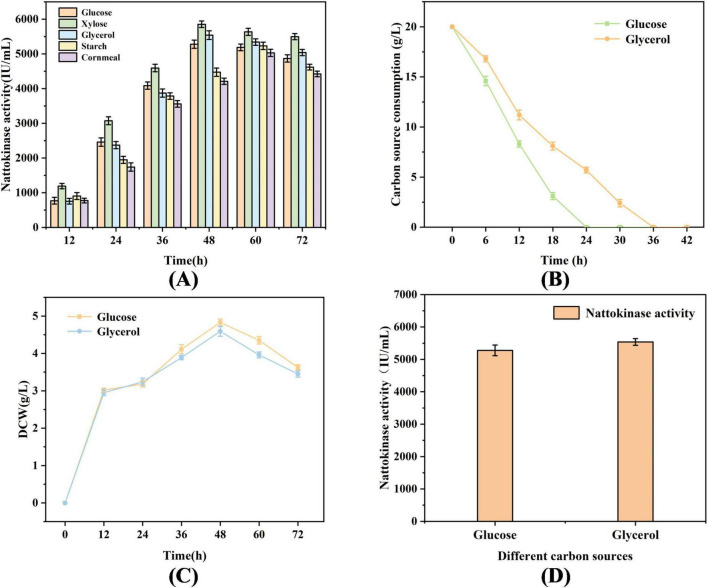
Biomass and nattokinase activity of *B. subtilis* 13,932 cultured with different carbon sources. **(A)** Fermentation of nattokinase activity by different carbon sources **(B)** Comparison of carbon source consumption of glycerol and glucose **(C)** Biomass comparison of glycerol and glucose fermented *B. subtilis* 13,932. **(D)** Highest nattokinase activity in glycerol and glucose fermentation.

As illustrated in Figure 1A comparative analysis was conducted to evaluate the differences in enzyme production for nattokinase fermentation using various carbon substrates. Notably, both glycerol and glucose demonstrated marked advantages over starch and cornmeal, with nattokinase activities measured at 5538.94 and 5278.63 IU/mL, respectively. However, it is lower than the nattokinase activity with xylose as a carbon source, and it is not used considering that the high price of xylose is not suitable for industrial production. It is noteworthy that in Figures 1B–D, in comparison to glucose, glycerol exhibited a relatively lower consumption rate and a reduced biomass dry weight. During the fermentation process, the decline in DCW after 48 h is primarily attributed to cell autolysis, accumulation of toxic metabolic byproducts, and changes in secondary metabolic regulation. There is a strong correlation between the reduction in DCW and the decrease in nattokinase activity.

### 3.2 Overexpression of glycerol metabolism gene

**FIGURE 2:**
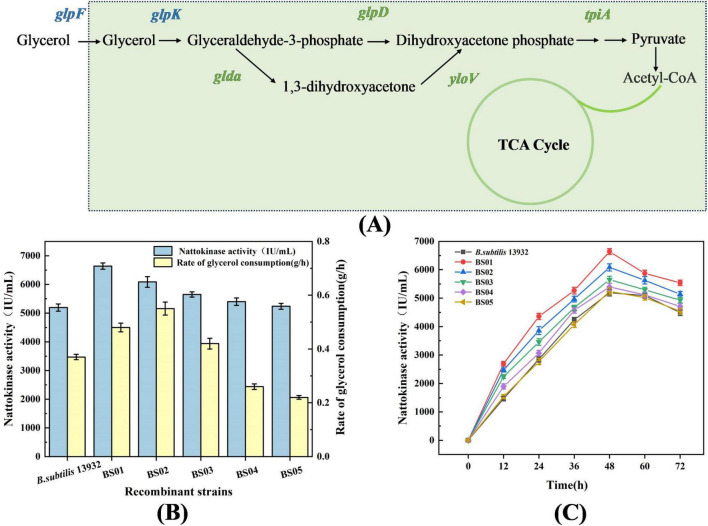
Effect of overexpression of glycerol metabolism pathway genes on glycerol utilization and nattokinase synthesis. **(A)** Schematic diagram of the intracellular glycerol metabolism pathway in *B. subtilis*. **(B)** Highest enzyme activity and glycerol consumption rate of recombinant strains. **(C)** Nattokinase activity of recombinant strains.

To enhance the utilization of glycerol and improve nattokinase production, *B. subtilis* 13,932 was engineered to overexpress key genes involved in glycerol metabolism, including *glpF, glpK, glpP, glpD*, and *tpiA*. The results, shown in Figure 2, indicate that overexpression of *glpF* (glycerol permease) and *glpK* (glycerol kinase) resulted in the most efficient glycerol utilization. Within 24 h in the glycerol fermentation medium, the residual glycerol in the two recombinant strains was completely consumed, with glycerol consumption rates of 0.48 and 0.55 g/h, respectively. Corresponding nattokinase activities for these strains were 6639.35 and 6085.74 IU/mL, compared to the control strain’s values of 0.37 g/h for glycerol consumption and 5494.57 IU/mL for nattokinase activity. These results suggest that overexpression of *glpF* and *glpK* significantly increased the efficiency of glycerol conversion to nattokinase. Previous studies have shown that *glpF* is crucial for enhancing glycerol uptake, while *glpK* is a rate-limiting enzyme in glycerol metabolism (Wang et al., 2022), confirming the importance of these two genes for efficient glycerol utilization. In addition to *glpF* and *glpK*, overexpression of the *glpP* gene, which encodes the glycerol uptake operon anti-terminator regulatory protein, also led to improved glycerol consumption, with a rate of 0.42 g/h. This strain exhibited the highest nattokinase activity (5649.28 IU/mL), as GlpP activates the expression of key glycerol metabolism genes by disrupting the terminator adjacent to the promoter (Darbon et al., 2002; Fujita, 2009). However, the overexpression of *glpD* (glycerol-3-phosphate dehydrogenase) and *tpiA* (triosephosphate isomerase) did not significantly improve glycerol utilization or nattokinase production, indicating that these genes are less critical for enhancing the metabolic flux through the glycerol pathway in this context (Beijer and Rutberg, 1992).

### 3.3 Combinational expression

**FIGURE 3:**
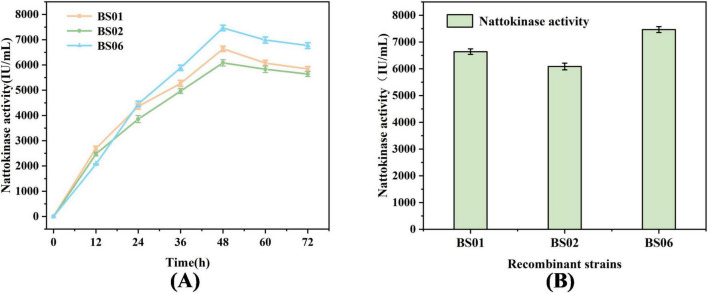
Effect of combined expression of *glpF* and *glpK* in the recombinant strain on nattokinase synthesis. **(A)** Comparison of fermented nattokinase activity of recombinant strains. **(B)** Highest nattokinase activity of the recombinant strain.

In previous experiments, we observed that overexpression of the *glpF* and *glpK* genes significantly increased the rate of glycerol consumption and enhanced nattokinase production. To further improve the conversion of glycerol to nattokinase, we constructed a recombinant strain, BS06, by combining both *glpF* and *glpK* genes, as shown in Figure 3A. The results in Figure 3B demonstrate that after 48 h of fermentation, glycerol was completely consumed, and nattokinase activity reached its peak. Specifically, the nattokinase activities for the recombinant strains BS01, BS02, and BS06 were 6639.35, 6086.74, and 7465.85 IU/mL, respectively. These findings indicate that the combined overexpression of *glpF* and *glpK* resulted in the highest nattokinase synthesis, confirming the effectiveness of this approach.

### 3.4 Response surface analysis for optimization

Based on relevant literature and experimental results, compounds such as glycerol, okara, MgSO_4_⋅7H_2_O, and CaCl_2_ have been shown to significantly enhance nattokinase activity (Chau, 2017; Chen et al., 2007; Deepak et al., 2008). To further optimize nattokinase production, a three-level Box-Behnken Design (BBD) experiment, considering four factors, was implemented using Design-Expert 13 software (Modi et al., 2022). A total of 29 experimental runs were performed, with each run repeated three times. The results of the variance analysis (shown in Table 4) indicate that the model’s F-value is 59.02, and the *p*-value is greater than 0.05, suggesting that the differences observed are not statistically significant. This implies that unknown factors had minimal impact on the experimental outcomes, and the fitted multivariate quadratic regression equation is reliable. The quadratic polynomial equation related to nattokinase activity is as follows:

**TABLE 4:** Analysis of variance for response surface quadratic model results.

Source	Sum of squares	df	Mean square	F-value	*P*-value	Significant
Model	2.545E + 07	14	1.818E + 06	59.02	< 0.0001	**
A-Glycerol	3.453E + 06	1	3.454E + 06	112.15	< 0.0001	**
B-Okara	1.631E + 06	1	1.631E + 06	52.95	< 0.0001	**
C-MgSO_4_⋅7H_2_O	2.438E + 05	1	2.438E + 05	7.92	0.0138	*
D-CaCl_2_	65599.57	1	65599.57	2.13	0.1665	–
AB	29131.66	1	29131.66	0.9459	0.3473	–
AC	304.15	1	304.15	0.0099	0.9222	–
AD	1.751E + 05	1	1.751E + 05	5.69	0.0318	*
BC	128.71	1	128.71	0.042	0.9494	–
BD	1982.48	1	1982.48	0.0644	0.8034	–
CD	7490.04	1	7490.04	0.2432	0.6295	–
A^2^	1.960E + 07	1	1.960E + 07	636.42	< 0.0001	**
B^2^	2.729E + 05	1	2.729E + 05	8.86	0.0100	*
C^2^	4.310E + 05	1	4.310E + 05	13.99	0.0022	*
D^2^	1.948E + 05	1	1.948E + 05	6.33	0.0247	*
Residual	4.312E + 05	14	30797.76	–	–	–
Lack of Fit	3.588E + 05	10	35879.63	1.98	0.2661	Not significant
Pure Error	72372.30	4	18093.07	–	–	–
Cor Total	2.588E + 07	28		–	–	–

*Indicates a significant difference (*p* < 0.05);

**Indicates an extremely significant difference (*p* < 0.0001).

Y = 8509.23 + 536.49 × A + 368.65 × B + 142.53 × C + 73.94 × D-85.34 × A × B + 8.72 × A × C-209.22 × A × D-5.67 × B × C-22.26 × B × D-43.27 × C × D-1738.30 × A^2-205.11 × B^2-257.76 × C^2-173.30 × D^2. The analysis revealed that the linear terms A, B, C, and D, as well as the cross-product term AC, had a positive effect on nattokinase activity, meaning they contributed to an increase in activity. In contrast, the cross-product terms AB, AD, BC, and BD, along with the quadratic terms A^2^, B^2^, D^2^, and E^2^, had a negative effect, resulting in a decrease in nattokinase activity.

**FIGURE 4:**
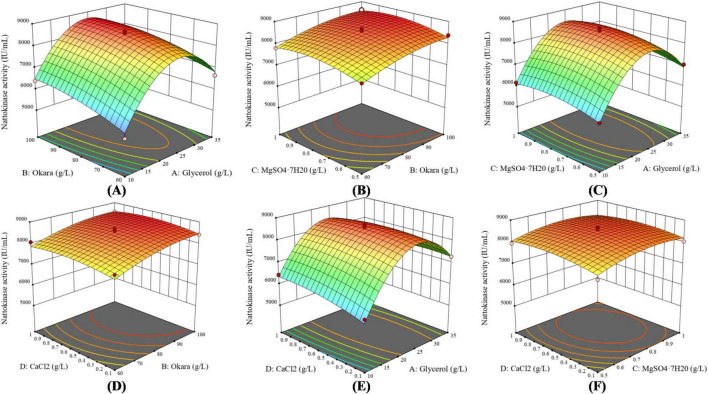
Response surface curves depicting the effects of medium components on nattokinase activity. The response curves illustrate the interactions between different factors: **(A)** Interaction between glycerol and soybean dregs (okara), **(B)** Interaction between MgSO_4_⋅7H_2_O and soybean dregs (okara), **(C)** Interaction between MgSO_4_⋅7H_2_O and glycerol, **(D)** Interaction between CaCl_2_ and soybean dregs (okara), **(E)** Interaction between CaCl_2_ and glycerol, and **(F)** Interaction between MgSO_4_⋅7H_2_O and CaCl_2_.

According to the variance analysis results in Table 4, the production of nattokinase was significantly affected by glycerol (*p* < 0.0001) and okara (*p* < 0.0001). As shown in Figure 4, increasing glycerol, MgSO_4_⋅7H_2_O, and CaCl_2_ initially increased nattokinase activity, which then decreased after reaching a peak. The 3D response surface analysis showed a convex pattern, indicating that nattokinase activity (y) reached a maximum within the selected variable ranges (x). Using Design-Expert 13 software, the optimal fermentation conditions for recombinant strain BS06, using glycerol as the carbon source and okara as the nitrogen source, were determined as follows: glycerol 22.97, okara 95.56, MgSO_4_⋅7H_2_O 0.80, and CaCl_2_ 0.71 g/L. Under these conditions, the predicted nattokinase activity was 8673.49 IU/mL. After adjusting for practical operability, the actual fermentation conditions were set to glycerol 23, okara 96, MgSO_4_⋅7H_2_O 0.8, and CaCl_2_ 0.7 g/L, resulting in a nattokinase activity of 8709.53 IU/mL. This slight increase over the predicted value further supports the reliability of the model.

### 3.5 Optimization of culture conditions

**FIGURE 5:**
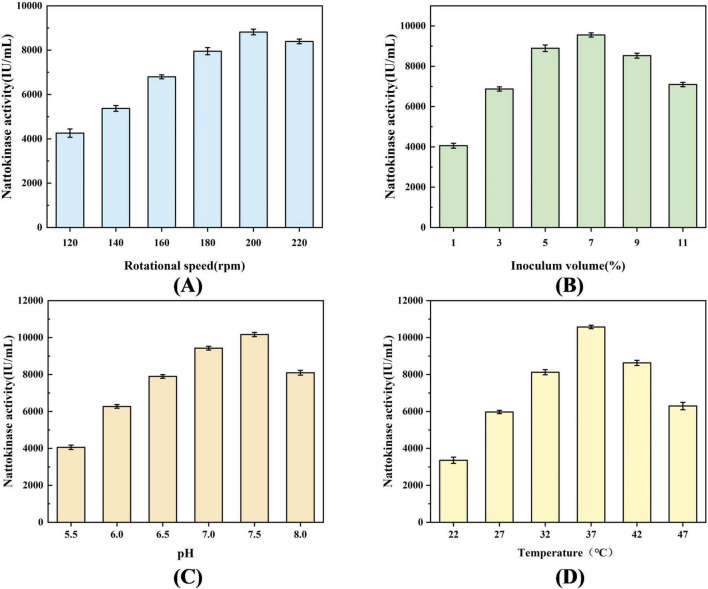
Effect of process parameters on nattokinase production by recombinant strains. **(A)** Effect of rotational speed on nattokinase production, **(B)** Effect of inoculum volume on nattokinase production, **(C)** Effect of pH on nattokinase production, and **(D)** Effect of temperature on nattokinase production.

Figure 5A shows the effect of different shaking speeds (120, 140, 160, 180, 200, and 220 rpm) on the nattokinase activity of recombinant strain BS06. The results indicated that nattokinase activity increased significantly with the shaking speed, reaching a peak value of 8817.48 ± 127.35 IU/mL at 200 rpm. However, when the shaking speed exceeded 200 rpm, the activity declined to 8394.37 ± 140.17 IU/mL. Figure 5B illustrates the impact of inoculum size (v/v) on enzyme activity. The highest nattokinase activity, 9553.07 ± 106.49 IU/mL, was observed at an inoculum size of 7%.

Figure 5C presents the effect of initial pH (5.5, 6.0, 6.5, 7.0, 7.5, and 8.0) on nattokinase activity (Khursade et al., 2018). The activity increased initially and then decreased with rising pH, with the highest enzyme activity of 10164.48 ± 114.26 IU/mL occurring at pH 7.5. Figure 5D shows the effect of temperature on nattokinase production. The enzyme activity gradually increased with temperature in the range of 22°C–37°C, reaching a peak of 10576.28 ± 91.78 IU/mL at 37°C. Above 37°C, enzyme activity began to decline.

Based on these findings, the optimal fermentation conditions for recombinant strain BS06 were determined through single-factor optimization. The optimal conditions were 37°C, 200 rpm shaking speed, 7% inoculum size (v/v), and an initial pH of 7.5. Under these conditions, the nattokinase activity reached a maximum of 10576.28 ± 91.78 IU/mL.

## 4 Discussion

The microbial transformation of renewable biomass resources, such as glycerol and okara, presents a promising avenue for advancing the circular economy (Ramamurthy et al., 2021; Tuly and Ma, 2024). By converting waste materials into valuable enzymes and platform chemicals, this process contributes to sustainability by reducing reliance on non-renewable carbon sources and minimizing environmental impact (Bishnoi et al., 2023; Chu et al., 2024; Eunji et al., 2024). This study presents a novel and sustainable approach for nattokinase production by utilizing glycerol and okara as cost-effective carbon and nitrogen sources. Glycerol, a byproduct of biodiesel production, and soybean residue, a waste material from tofu processing, were employed as renewable substrates, offering an eco-friendly alternative to conventional resources such as glucose and soybean peptone. Through metabolic engineering, the overexpression of key glycerol metabolic genes (*glpF* and *glpK*) significantly enhanced glycerol utilization efficiency and nattokinase yield. This strategy aligns with the trend in biomanufacturing that emphasizes the integration of low-cost raw materials and metabolic pathway optimization to improve production efficiency and sustainability. The findings underscore the potential of combining renewable resources with advanced metabolic engineering techniques to achieve economically viable and environmentally friendly nattokinase production. This approach not only reduces production costs but also contributes to waste management and the development of a circular economy. Future research should focus on exploring additional low-cost substrates and refining metabolic engineering strategies to further optimize the production process.

In this study, glycerol and okara were utilized as carbon and nitrogen sources, respectively, for nattokinase production. Our optimization experiments showed that glycerol, as a carbon source, significantly enhanced nattokinase synthesis, aligning with predictions from Pornkamol Unrean et al. (Unrean, 2013). Who demonstrated that sufficient glycerol and oxygen are optimal for high-yield nattokinase production. In terms of genetic modification, previous studies have attempted to enhance glycerol utilization by overexpressing key genes involved in glycerol metabolism, such as *glpK* and *glpF*. However, these studies have largely focused on the overexpression of single genes, failing to fully explore the potential of synergistic effects among multiple genes. For instance, Wang et al. (2022) improved the catalytic performance of glycerol kinase through chromosomal site-directed mutagenesis, yet their research did not address the synergistic role of glycerol permease (*glpF*), resulting in limited enhancement of glycerol utilization efficiency. Furthermore, significant research gaps remain in understanding how genetic modifications can be further optimized to increase nattokinase production. Our results further indicate that the combined expression of key glycerol metabolism genes (*glpFK*) in *B. subtilis* accelerated glycerol consumption and slightly improved nattokinase yield, addressing the aforementioned research gaps that demonstrate the benefits of metabolic engineering in enhancing substrate utilization (Applebee et al., 2011; Schweizer et al., 1997). However, given that the overexpression of multiple genes may impose a metabolic burden, leading to resource competition, metabolic imbalance, and cellular stress responses, a balanced approach is essential. To address these challenges, strategies such as stepwise optimization, dynamic regulation systems (e.g., inducible promoters), and metabolic pathway balancing (e.g., knockout of competing pathways) can be employed to alleviate metabolic burden and maximize target product synthesis. By integrating transcriptomic and metabolomic analyses with fermentation performance evaluation, strain performance can be systematically optimized to enhance nattokinase production while maintaining cellular metabolic homeostasis. Additionally, okara, a by-product of soybean processing with a crude protein content of 15.2%–33.4% (Li et al., 2023), proved to be an effective nitrogen source for nattokinase production, supporting its potential as a low-cost alternative to traditional nitrogen sources. For example, Li et al. (2022) successfully used okara to ferment *Bacillus natto*, producing both nattokinase and amylase, with enzyme activities of 1633.9 IU/mg and 39.93 U/mg, respectively. These findings underscore the potential of using agricultural waste in biotechnology processes.

Further optimization of the fermentation medium using glycerol, okara, MgSO_4_⋅7H_2_O, and CaCl_2_ resulted in a nattokinase activity of 8709.53 IU/mL. This demonstrates that glycerol and okara are viable, cost-effective substrates for high-yield nattokinase fermentation. By integrating renewable biomass into the production of biochemicals like nattokinase, we not only enhance enzyme synthesis but also contribute to the circular economy by reducing waste and promoting sustainable resource use.

## 5 Conclusion

The fermentation of renewable biomass for the production of enzymes and chemicals represents a promising and sustainable approach. In this study, the overexpression of the glycerol-utilizing metabolic gene *glpFK* was found to significantly enhance nattokinase activity, achieving a maximum value of 7465.85 IU/mL. Concurrently, soybean dregs, a by-product of tofu processing, were used as a nitrogen source for nattokinase fermentation. Through response surface methodology and subsequent optimization of fermentation conditions, the nattokinase activity was further increased, reaching a peak value of 10576.28 ± 91.78 IU/mL. These findings suggest that the combined use of glycerol as a carbon source and soybean dregs as a nitrogen source constitutes an effective and sustainable strategy for the production of nattokinase.

## Data Availability

The original contributions presented in this study are included in this article/supplementary material, further inquiries can be directed to the corresponding author.
